# Evolutionary history influences the salinity preference of bacterial taxa in wetland soils

**DOI:** 10.3389/fmicb.2015.01013

**Published:** 2015-10-02

**Authors:** Ember M. Morrissey, Rima B. Franklin

**Affiliations:** Laboratory of Microbial Ecology, Department of Biology, Virginia Commonwealth UniversityRichmond, VA, USA

**Keywords:** phylogeny, biogeochemistry, saltwater intrusion, methanogen, marsh, community composition

## Abstract

Salinity is a major driver of bacterial community composition across the globe. Despite growing recognition that different bacterial species are present or active at different salinities, the mechanisms by which salinity structures community composition remain unclear. We tested the hypothesis that these patterns reflect ecological coherence in the salinity preferences of phylogenetic groups using a reciprocal transplant experiment of fresh- and saltwater wetland soils. The salinity of both the origin and host environments affected community composition (*16S rRNA* gene sequences) and activity (CO_2_ and CH_4_ production, and extracellular enzyme activity). These changes in community composition and activity rates were strongly correlated, which suggests the effect of environment on function could be mediated, at least in part, by microbial community composition. Based on their distribution across treatments, each phylotype was categorized as having a salinity preference (freshwater, saltwater, or none) and phylogenetic analyses revealed a significant influence of evolutionary history on these groupings. This finding was corroborated by examining the salinity preferences of high-level taxonomic groups. For instance, we found that the majority of α- and γ-proteobacteria in these wetland soils preferred saltwater, while many β-proteobacteria prefer freshwater. Overall, our results indicate the effect of salinity on bacterial community composition results from phylogenetically-clustered salinity preferences.

## Introduction

Understanding if and how the evolutionary history of a species relates to its ecology is a fundamental question for biologists. For macroorganisms, phylogeny is often an ecologically meaningful way to classify organisms, as closely related taxa frequently have similar ecological characteristics (e.g., Silvertown et al., 2006; Donoghue, 2008) and functional traits (e.g., Cavender-Bares et al., 2009). In contrast, the phylogeny of microorganisms is generally considered to be an unreliable indicator of ecology because microbes can evolve quickly (Vasi et al., 1994) and engage in horizontal gene transfer (Snel et al., 2002). Nonetheless, most research into the ecology of microorganisms has relied on phylogenetic classification, and there is an accumulating body of evidence that shows phylogenetically-clustered taxa exhibit a substantial degree of ecological similarity, even at high levels of taxonomic organization (Philippot et al., 2010; Langille et al., 2013). For instance, ecological coherence [when members of a phylogenetic group share strategies or traits that distinguish them from other clades (Philippot et al., 2010)] has been demonstrated for the orders of α-proteobacteria, where members of each order are similar in multiple regards including habitat preference and genome size (Ettema and Andersson, 2009). However, there has been virtually no research into whether the responses of bacterial phylotypes to key environmental variables are phylogenetically conserved. An increased understanding of these relationships could lead to a more predictive understanding of how environmental variables shape bacterial community composition and could help explain global patterns in bacterial biodiversity.

Salinity has been proposed to be a major driver of phylogenetic bacterial community composition across the planet (Lozupone and Knight, 2007), and numerous studies in aquatic systems have found the relative abundance of high-level taxonomic groups (e.g., Phylum, Class) to correlate with salinity (e.g., del Giorgio and Bouvier, 2002; Herlemann et al., 2011). Though it has not yet been explored, one explanation for these patterns is that they reflect ecological coherence of salinity preferences. An increased understanding of the mechanisms by which salinity influences microbial communities is especially important given the widespread ongoing salinization of coastal habitats due to climate change and anthropogenic modification of the hydrologic cycle (Herbert et al., 2015). Tidal freshwater wetlands are particularly vulnerable, and sea-level rise is expected to cause saltwater intrusion into these ecosystems (Neubauer and Craft, 2009). To date, the biogeochemical responses of these systems to elevated salinity have been incongruous. For example, thermodynamic considerations have led many scientists to predict that methanogenesis, the dominant anaerobic pathway in organic matter degradation in tidal freshwater wetland soils, will be suppressed as salinity increases. This expectation is based on the fact that the elevated sulfate concentrations associated with more saline water allow sulfate-reducing bacteria (SRB) to outcompete methanogens. There are several reports in the literature that are consistent with this expectation (e.g., Weston et al., 2006; Chambers et al., 2011; Neubauer, 2013) but also some that are not (Weston et al., 2011; Hopfensperger et al., 2014). Along these same lines, increases in salinity have been reported to both increase (Weston et al., 2006; Craft, 2007) and decrease (Roache et al., 2006; Neubauer et al., 2013) decomposition rates in wetlands. These discordant responses may be driven by differences in the composition of the underlying microbial communities and their dynamic responses to altered salinity, which may be phylogenetically conserved. Thus, characterizing the salinity preferences of phylogenetic groups will enhance our ability to predict how a change in salinity will affect a given microbial community and its associated functions.

The current study was designed to: (i) test whether salinity preferences exhibit phylogenetic conservation, and (ii) characterize the response of wetland microbial community composition and function to changes in salinity. To address these goals, we performed a reciprocal transplant experiment of fresh- and saltwater wetland soils and monitored phylogenetic community structure (bacterial *16S rRNA* gene sequences) and activity (extracellular enzyme activity, CO_2,_ and CH_4_ production).

## Materials and Methods

### Experimental Design

This research was conducted in two tidal wetlands (Cumberland and Taskinas Creek marshes) on the Pamunkey River, a major tributary of the Chesapeake Bay in Virginia (USA). The two marshes are close enough in proximity that weather, land-use, and underlying lithology do not differ, but a strong salinity gradient means that Cumberland Marsh (37.548, -76.984) is freshwater (ppt <0.5) while Taskinas Marsh (37.414, -76.717) is brackish (4–15 ppt). When this study was conducted, the dominant vegetation at Cumberland marsh was *Peltandra virginica* whereas *Spartina alterniflora* dominated Taskinas. In each marsh, a 10 m × 10 m plot was established within which 10 soil cores (10-cm diameter, 10-cm depth; 785 cm^3^ volume) were randomly collected. Each core was transferred into a chamber made of PVC pipe (10-cm diameter, 10-cm height) with PVC caps secured on the top and bottom. Caps had 8.25-cm diameter holes drilled in them, and these openings were covered with two layers of 0.5-mm Nitex mesh (Wildlife Supply Company, Buffalo, NY, USA) secured with Amazing Goop (Eclectic Products, Eugene, OR, USA). After soil cores were placed in the chambers, the caps were sealed to the PVC cylinder using Amazing Goop. The next morning, chambers were returned the marsh for *in situ* incubation. Each chamber was gently inserted into one of the holes created the day before (when cores were collected); location assignments within each plot were random. The reciprocal transplant generated four treatments with five replicates of each combination of “soil origin” and “host site” to yield: Fresh–Fresh (soil originating from the freshwater site, incubated in the freshwater site), Fresh–Salt, Salt–Salt, and Salt–Fresh. Chambers were incubated for 40 days from June–August 2013. When chambers were retrieved, each was sealed in an air-tight plastic bag and immediately returned to the laboratory for analysis.

### Soil Characterization

Soil salinity (ppt) and pH were measured using a SG78 SevenGo Duo pro probe (Mettler Toldeo, Columbus, OH, USA). Soil moisture (%) was determined gravimetrically (100°C for 72 h) and organic matter (OM, %) was measured as mass loss on ignition (500°C for 4 h). Total carbon and nitrogen contents were determined using a Perkin Elmer CHNS/O Analyzer (Waltham, MA, USA) following grinding and acidification of samples using 10% hydrochloric acid; C:N was calculated by mass. Porewater was extracted from 50-ml soil samples by centrifugation (3000 × *g*, 15 min), filtered using a 0.45 μm pore-size mixed cellulose ester syringe filter, and stored at -20°C until it could be analyzed for sulfate concentration via ion chromatography (Dionex ICS-1000, Sunnyvale, CA, USA).

### Molecular Genetic Analyses

Whole-community DNA was extracted from 0.5-g subsamples of soil using the MoBio PowerSoil DNA Isolation Kit (Carlsbad, CA, USA) and stored at -20°C. DNA purity and concentration were analyzed using a Nanodrop ND-1000 (Thermo Scientific, Wilmington, DE, USA). All DNA extracts and PCR products were verified using agarose gel (1.5%) electrophoresis and ethidium bromide staining.

Bacterial *16S rRNA* and methanogen *mcrA* gene abundances were assessed using quantitative PCR (qPCR) as described in Morrissey et al. (2014a). Briefly, the primers Eub338 and Eub517 were used to target the *16S rRNA* gene (average efficiency = 99.7%, *r*^2^ = 0.99), and the mlas and mcrA-rev primers were used to target the methyl coenzyme-M reductase encoding the *mcrA* functional gene (average efficiency = 90.0%, *r*^2^ = 0.99). A third qPCR was employed to quantify the abundance of the *dsrA* gene using the primers dsrA_290F and dsrA_660R designed by Pereyra et al. (2010). These primers target the alpha subunit of the dissimilatory sulfite reductase gene of δ-proteobacterial SRB. Reactions (20 μl) were performed with 6 ng DNA template and 0.2 μM concentrations of each primer; thermal cycling conditions were: 95°C for 10 min, and 50 cycles of 40 s at 95°C, 30 s at 60°C, and 30 s at 72°C. *Desulfovibrio desulfuricans* (Strain 27774, ATCC, Manassas, VA, USA) was used to establish the standard curve (average efficiency = 93.4%, *r*^2^ = 0.99). For all three genes, qPCR assays were performed using SsoAdvanced SYBR Green qPCR Supermix (BioRad, Hercules, CA, USA) and a BioRad CFX 96 Real-Time System. Data were analyzed using Bio-Rad CFX Manager Version 2.1. Results were reported as the log_(10)_ of the number of gene copies per g of OM after averaging three technical replicates per sample.

Composition of the bacterial community was assessed using Illumina HiSeq sequencing of the hyper variable V3 region of the *16S rRNA* gene using the primers and protocol described by Bartram et al. (2011). Briefly, PCR reactions (50 μl) were performed in triplicate for each sample using 10 mM TrisHCl (pH 8.3), 50 mM KCl, 200 μM of each dNTP, 20 μg BSA, 2.5 units of AmpliTaq DNA polymerase, 0.5 μM of each primer, 1.5 μM MgCl_2,_ and 4 ng DNA template (reagents obtained from Applied Biosystems, Foster City, CA, USA). Thermal cycling conditions were: 95°C for 5 min, 20 cycles of 60 s at 95°C, 60 s at 50°C, 60 s at 72°C, followed by 72°C for 7 min (PTC-100 Thermal Controller, MJ Research, Waltham, MA, USA). Triplicate PCR products were pooled and purified using AMPure XP (Agencourt-Beckman, Brea, CA, USA) prior to sequencing on the Illumina Hiseq (at VCU’s Nucleic Acid Research Facility) using 2 × 300 bp read chemistry. Quality filtered reads (Phred score ≥ 30 indicating 99.9% base call accuracy) were received as pairs of demultiplexed fastq files; the length of the locus was within the high quality region of read one, so the second read was not used. Sequence and accompanying metadata are available on the MG-RAST server (Meyer et al., 2008) project number 12548.

### Extracellular Enzyme Activity

Within one week following sample collection, soils were assayed for extracellular enzyme activity (EEA) using the fluorimetric and colormetric microplate assays described in Neubauer et al. (2013) and a Synergy 2 plate reader (Biotek, Winooski, VT, USA). Briefly, enzyme activity associated with breakdown of cellulose [β-1,4-glucosidase (BG) and 1,4- β-cellobiosidase (CBH)], hemicellulose [β-D-xylosidase (BX)], and lignin [phenol oxidase (POX) and peroxidase (PR)], as well as the release of nitrogen [leucyl aminopeptidase (LAP)] and sulfur [arylsulfatase (AS)] from organic molecules, was measured. Fluorescent assays relied on the following substrates: 4-MUB β-D-glucopyranoside (BG), 4-MUB β-D-cellobioside (CBH), 4-MUB- β-D-xylopyranoside (BX), L-leucine-7-AMC (LAP), and 4-MUB-sulfate (AS) and followed incubations of either 1 (CBH, LAP) or 4 h (BG, BX, AS) at 30°C with gentle agitation. POX and PR activities was measured colorimetrically via the oxidation of l-DOPA (in the presence of H_2_O_2_ for PR) after a 30 min incubation using the methods of Sinsabaugh et al. (2003). Substrates and reagents were obtained from Sigma–Aldrich Co. Ltd (St. Louis, MO, USA).

### Anaerobic CO_2_ and CH_4_ Production

Production of CO_2_ and CH_4_ was measured using 48-h anaerobic slurry assays (after Neubauer et al., 2005; Morrissey et al., 2014a) employing two technical replicates per sample. Gas samples (5 ml) were obtained from the headspace of each slurry after 0, 22, 32, and 48 h, and later run on a GC-2014 gas chromatograph equipped with an flame ionization detector, thermal conductivity detector, electron capture detector, and methanizer (Shimadzu; Columbia, MD, USA). For each gas, total production was determined as the sum of the gas accumulated in the headspace and the gas dissolved in the slurry; the later was estimated using the relationships described by Weiss (1974). Gas concentrations increased linearly over time, and production rates (ng C g OM^-1^ h^-1^) were calculated using linear regression; median correlation coefficients were 0.87 for CO_2_ and 0.92 for CH_4_.

### Data Analysis

All environmental, enzyme activity, and gas production data were normally distributed within each treatment; microbial gene abundances (*16S rRNA*, *mcrA*, *dsrA*) required a log_(10)_ transformation prior to statistical analysis. Effects of host and origin environments on environmental and functional parameters as well as microbial gene abundances were assessed using two-way analysis of variance (ANOVA, *n* = 5 per group, df = 19) with Tukey’s HSD for *post hoc* comparisons. Similarly, the effect of origin and host environment on the relative abundance of putative sulfate reducing δ-proteobacteria (Zhou et al., 2011), putative iron reducing bacteria (FeRB, specifically *Geobacter* (Mahadevan et al., 2011), and other phylogenetic groups identified from the Illumina data, were analyzed using two-way ANOVA on log transformed values. Relationships between microbial gene abundances were assessed using Pearson correlations. These analyses were performed in the JMP (JMP Pro 10, Cary, NC, USA; Sall et al., 2005) with α = 0.05. A Mantel test was used to compare microbial activity (Gower distance using EEA and gas production) with bacterial community composition (weighted UniFrac distance) in PAST (Hammer, 2001).

Bacterial *16S rRNA* sequences were clustered into operational taxonomic units (OTUs, a.k.a. phylotypes) using 97% sequence identity in Qiime 1.7 using default parameters (Caporaso et al., 2010). OTUs accounting for less than 0.005% of sequences were removed prior to downstream analyses (Bokulich et al., 2013). The number of reads per sample varied from 21,398 to 122,666. Median read depth was 58,959 sequences per sample, and samples were rarified to 21,398 sequences per sample for diversity analyses. Because the sequences were too short for accurate construction of a phylogenetic tree, the representative sequence for each OTU was blasted against the Greengenes database version 13_8 using Qiime (Edgar, 2010; McDonald et al., 2012). The reference sequence with the greatest percent identity match for each OTU was used for phylogenetic assignment and downstream analyses; median percent identity was 97.8%. The Greengenes 97% OTU tree was pruned to contain only the OTUs present in our samples for phylogenetic analysis. The resultant OTU table and phylogenetic tree were then analyzed in R version 3.1.2.

Beta diversity (weighted UniFrac distances; Lozupone and Knight, 2005) was visualized using principal coordinates analysis (PCoA) with the ape package (Paradis et al., 2004) and analyzed via PerMANOVA with the vegan package (Oksanen et al., 2013). A phylotype was deemed “present” in a treatment if it was found in the majority of sample replicates (i.e., at least three of five), and otherwise considered “absent” (after Evans and Wallenstein, 2014). This presence/absence data was used to create a Venn diagram using the venneuler package (Wilkinson, 2012). Habitat preference was inferred by comparing the presence/absence patterns of OTUs across the treatments. First, phylotypes native to both the fresh and saltwater sites (i.e., present in both the Fresh–Fresh and Salt–Salt treatments) were considered to exhibit no strong salinity preference. Next, phylotypes with a preference for freshwater were identified as ones that fit the following criteria : (a) present in the freshwater controls (Fresh–Fresh), (b) present in the soils of saltwater origin following transplantation to the freshwater environment (Salt–Fresh), and (c) absent in the saltwater controls (Salt–Salt). Similarly, a preference for saltwater was defined as those phylotypes that were: (a) present in the saltwater controls (Salt–Salt), (b) present in soils of freshwater origin after transplantation to the saltwater environment (Fresh–Salt), and (c) absent in the freshwater controls (Fresh–Fresh). This presence/absence criterion does not assign salinity preferences to phylotypes that were simply enriched or depleted (as determined by changes in *16S rRNA* relative abundance) in the different salinity environments. Consequently, the “no preference” category may contain organisms that favor one salinity over the other but can survive in both environments. We also recognize that the OTUs we classified as “absent” could still be present in the community, only their abundance is below our detection limit. Overall, our characterization using presence/absence criterion is a conservative approach designed to identify strong salinity preferences. After each phylotype was assigned a salinity preference, the standardized effect size of phylogenetic diversity (SES-PD) and the nearest taxon index (NTI) were calculated for the salinity preference groups and examined to see if these indices reflected more or less phylogenetic relatedness than would be expected by chance using the ses.pd and ses.mntd functions, respectively, in the picante package (Kembel, 2009). Bootstrapping with 6000 iterations using the bootstrap package (Efron and Tibshirani, 1993) was used to produce 95% confidence intervals for the % of phylotypes with each salinity preference in the different bacterial clades. If the confidence intervals did not overlap with the expected random value (i.e., the % of total phylotypes in that preference group), those clades were considered to have more or less phylotypes in a preference category than would be expected by chance.

## Results

### Environment

Soils originating from the freshwater site had lower pH but higher moisture content, OM, and C:N than the soils originating from the saltwater site (**Table 1**). None of these parameters changed significantly upon transplant, except that the pH of the soil from the saltwater site decreased. As expected, the host environment regulated the soil porewater salinity and sulfate concentrations, wherein soils hosted in the saltwater site had greater solute concentrations than their freshwater counterparts (mean by host; salinity: 4.4 vs. 0.2 ppt, sulfate: 910 vs. 361 ppm).

**Table 1:** Mean ± SE of environmental, microbial abundance, extracellular enzyme, and gas production parameters for each treatment.

Parameters		Origin–Host				ANOVA
		Fresh–Fresh	Fresh–Salt	Salt–Fresh	Salt–Salt		
Environmental							
	Moisture (%)	72.1 ± 1.1	71.7 ± 0.9	64.9 ± 1.5	60.6 ± 0.9	O
	OM (%)	24.4 ± 1.0	26.5 ± 0.9	17.5 ± 1.2	15.1 ± 0.5	I
	C:N	14.5 ± 0.4	14.8 ± 0.2	13.3 ± 0.5	12.5 ± 0.2	O
	pH	5.9 ± 0.1	6.1 ± 0.1	7.4 ± 0.1	6.7 ± 0.1	I
	Salinity (ppt)	0.09 ± 0.01	4.09 ± 0.38	0.35 ± 0.11	4.70 ± 0.08	H
	Sulfate (ppm)	256 ± 108	1001 ± 380	467 ± 87	820 ± 144	H
Abundance^1^							
	Bacterial (*16S rRNA*)	10.1 ± 0.1	9.9 ± 0.1	10.0 ± 0.1	10.2 ± 0.1	n.s.
	Methanogen (*mcrA*)	9.4 ± 0.1	9.3 ± 0.1	7.9 ± 0.2	6.9 ± 0.1	I
	Sulfate reducers (*dsrA*)^2^	8.4 ± 0.2	8.3 ± 0.1	9.1 ± 0.1	9.2 ± 0.1	O
Enzyme activity^3^							
	BG	6129 ± 1324	5061 ± 1187	811 ± 136	882 ± 65	O
	CBH	179 ± 32	186 ± 26	171 ± 15	169 ± 38	n.s.
	LAP	1723 ± 159	1322 ± 309	3197 ± 312	3786 ± 172	O
	AS	408 ± 68	367 ± 67	1164 ± 136	1080 ± 43	O
	BX	613 ± 63	685 ± 92	627 ± 22	804 ± 54	n.s.
	POX	0.28 ± 0.03	0.23 ± 0.02	0.31 ± 0.03	0.35 ± 0.03	O
	PR	0.25 ± 0.03	0.17 ± 0.01	0.38 ± 0.03	0.28 ± 0.04	O
Gas production^4^							
	CO_2_	1339 ± 84	1071 ± 40	2512 ± 266	2066 ± 144	O,H
	CH_4_	3.42 ± 0.48	0.16 ± 0.01	0.52 ± 0.10	0.37 ± 0.06	I

*^1^log gene copies g OM^-1^.*

*^2^Deltaproteobacterial sulfate reducers.*

*^3^pmole substrate g OM^-1^ h^-1^.*

*^4^ng C g OM^-1^ h^-1^.*

*Two-way ANOVA results (α = 0.05) are presented as follows: “O”, main effect of origin environment; “H”, host environment; “I”, interaction between origin and host; and “n.s.”, not significant.*

### Microbial Community

#### Abundance

Bacterial (*16S rRNA*) abundance was not significantly different across the treatments (**Table 1**). In contrast, both the abundance of methanogens (*mcrA*) and SRB (δ-proteobacteria clade, *dsrA*) were affected by the origin of the soil, wherein the freshwater soils had higher methanogen abundance and lower SRB abundance. An interaction effect was observed for methanogens, wherein abundance in soils originating from the saltwater site increased upon transplantation to the freshwater environment. Bacterial abundance was not correlated with that of methanogens (*r* = -0.21, *p* = 0.36) or that of δ-proteobacterial SRB (*r* = 0.31, *p* = 0.17). However, a strong negative relationship was found between the abundance of methanogens and δ-proteobacterial SRB (*r* = -0.79, *p* < 0.01).

#### Bacterial Community Structure

**FIGURE 1:**
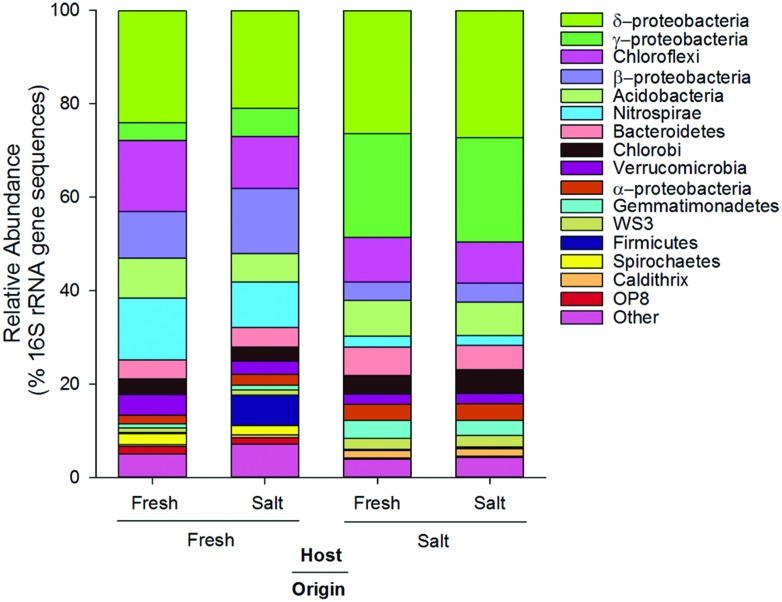
**Average relative abundance (% of *16S rRNA* gene sequences) of dominant phylogenetic groups (>3.3% of total sequences) in soils originating from and hosted in freshwater (Fresh) and saltwater (Salt) wetlands (*n* = 5 per treatment)**.

**FIGURE 2:**
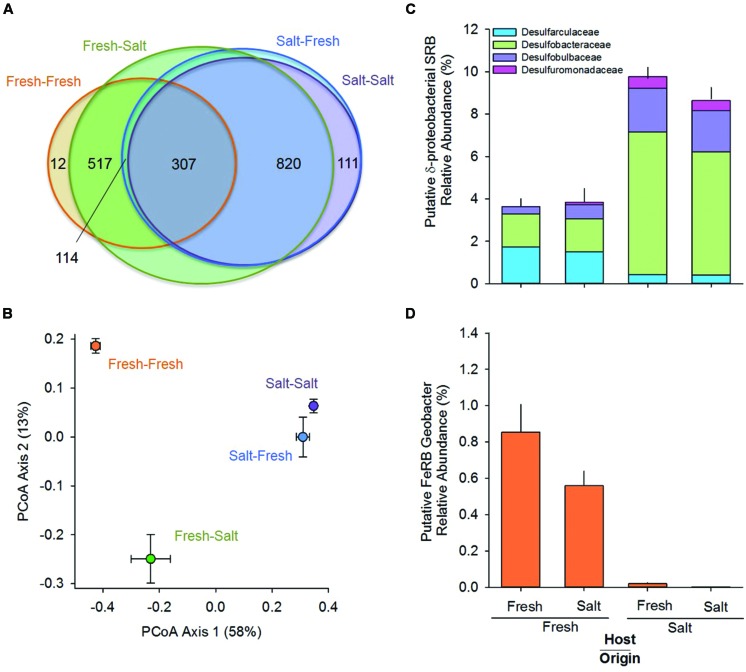
**Venn diagram showing the shared phylotypes (A); graph of the first two axes of a principal coordinates analysis (PCoA) prepared using weighted UniFrac distances to compare phylogenetic community composition (centroids are mean ± SE, *n* = 5 per treatment; B); and relative abundance of putative sulfate [sulfate-reducing bacteria (SRB; C)] and iron reducing bacteria [FeRB (D)] originating from and hosted in freshwater (Fresh) and saltwater (Salt) wetlands (*n* = 5 per treatment, mean and SE).** Please note, the Venn diagram represents an imperfect best-fit representation of the data, and phylotype membership is not labeled for all sectors.

In terms of relative abundance (% of *16S rRNA* gene sequences), all soils were dominated by Proteobacteria (specifically α, β, δ, and γ), Chloroflexi, Acidobacteria, and Bacteroidetes (**Figure 1**). However, soils of freshwater origin had higher relative abundances of β-proteobacteria (ANOVA, Origin, *F* = 64.9, *p* < 0.01) and Nitrospirae (ANOVA, Origin, *F* = 84.0, *p* < 0.01), while soils originating from the saltwater site had greater relative abundances of γ-proteobacteria (ANOVA, Origin, *F* = 107.1, *p* < 0.01). Saltwater control soils (Salt–Salt) had greater phylotype richness than freshwater control soils (Fresh–Fresh), and transplantation into an alternate salinity increased the phylotype richness of soils originating from both environments (**Figure 2A**). Phylogenetic community structure (assessed as weighted UniFrac distances) was interactively affected by both origin and host environments as determined by two-way PerMANOVA (Origin, *F* = 32.6; Host, *F* = 4.9; Origin × Host, *F* = 5.7, all *p* < 0.01). Though each community shifted when transplanted to the contrasting salinity environment, they remained more closely related to their origin environment controls than their host environment controls (**Figure 2B**).

The relative abundance and composition of putative SRB in the δ-proteobacteria clade differed between soils originating from the freshwater and saltwater environments (**Figure 2C**; two-way PerMANOVA of composition: Origin, *F* = 110.35, *p* < 0.01). Soils of freshwater origin had lower relative abundance of SRB (ANOVA, Origin, *F* = 75.4, *p* < 0.01) and a greater fraction of Desulfarculaceae, while SRB in soils of saltwater origin were dominated by the Desulfobacteraceae, Desulfobulbaceae, and Desulfuromonadaceae families. Both origin and host environment salinity influenced the relative abundance of the putative FeRB *Geobacter* (**Figure 2C**, ANOVA, Origin, *F* = 398.8; Host, *F* = 20.6; Origin × Host *F* = 7.4, all *p* < 0.02); *Geobacter* relative abundance was higher in soils originating from and or hosted in the freshwater environment.

#### Salinity Preference

**FIGURE 3:**
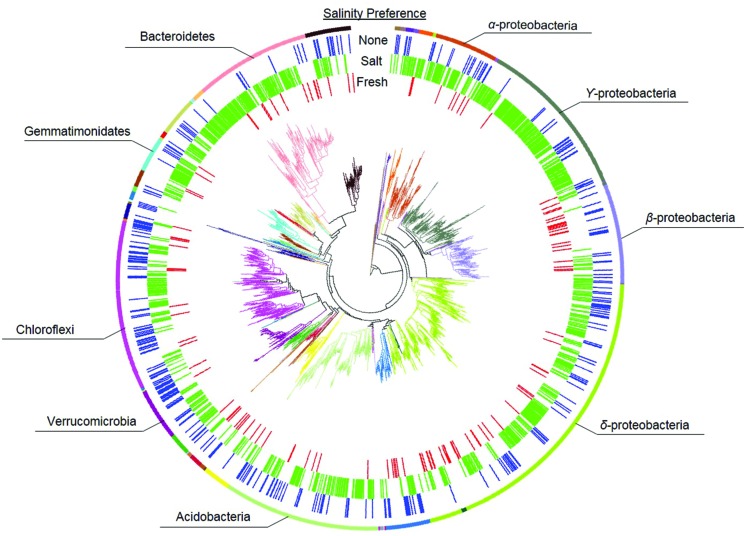
**Phylogeny of bacterial phylotypes in relation to salinity preference.** Phylogenetic groups are designated by branch color, and dominant groups are annotated. The inner three rings indicate salinity preferences as freshwater (red), saltwater (green), or no preference (none, blue).

Habitat preference with respect to salinity was discerned by comparing the presence/absence of phylotypes in the different treatments as described in the Section “Materials and Methods.” There were 114 phylotypes that were categorized as having a preference for freshwater, which means they were detected in soils incubated at the freshwater site (Salt–Fresh treatment and Fresh–Fresh controls) but were not found in the Salt–Salt treatment. Another 820 phylotypes were categorized as having a preference for saltwater by virtue of the fact that they were found in both soils incubated at the saltwater site (Fresh–Salt treatment and Salt–Salt controls), but not the Fresh–Fresh controls. The 307 phylotypes present in both the fresh and saltwater controls were categorized as having no salinity preference (**Figure 2A**). These salinity preferences were then compared to phylogeny to assess whether evolutionary history influenced habitat preference (**Figure 3**). The phylotypes in these categories were more closely related to each other than would be expected by chance as assessed by both the standardized effect size of phylogenetic diversity (SES-PD) and NTI. The strongest influence of phylogeny was seen in the phylotypes that had no salinity preference (SES-PD = -7.1, NTI = 6.5, both *p* < 0.01), followed by the saltwater (SES-PD = -5.4, NTI = 5.2, both *p* < 0.01) and freshwater (SES-PD = -3.5, NTI = 4.3, both *p* < 0.01) preference categories. Phyla varied with respect to their salinity preferences (**Figure 3**; Supplemental Figure S1). For instance, α- and γ-proteobacteria had more phylotypes with a preference for saltwater than would be expected by chance, while β-proteobacteria had more phylotypes that preferred freshwater, and Verrucomicrobia had more phylotypes with no preference than would be expected at random.

### Microbial Activity

As was observed for community composition, microbial activity appears to be most strongly controlled by the origin of the soil sample (**Table 1**). Overall, this variation in function was strongly correlated to variation in bacterial community structure (Mantel *R* = 0.76, *p* < 0.01).

Analysis of EEA revealed no treatment effects for either CBH or BX (**Table 1**). Soils of freshwater origin had a greater activity of BG (∼6.5-fold), AS (∼2.9-fold), and LAP (∼2.3-fold). In contrast, soils with a saltwater origin had a greater activity of POX (∼1.3-fold) and PR (∼1.6-fold). The only enzyme activity affected by the host environment was PR, which was ∼30% higher when hosted in the freshwater site.

Production of CO_2_ was heavily influenced by origin environment, where soils from the saltwater site exhibited higher rates (∼1.8-fold) than those from the freshwater site. Host environment had a modest but significant effect wherein the soils incubated at the freshwater site had rates of CO_2_ production that were ∼20% higher than their saltwater counterparts (**Table 1**). The production of CH_4_ was interactively regulated by soil origin and host environment wherein rates were very low in all treatments except for the soils originating from, and hosted in, the freshwater environment.

## Discussion

In this study, we investigated the effects of salinity on wetland microbial community composition and activity via an *in situ* salinity manipulation experiment, and then assessed whether phylogenetic clades of bacteria exhibited ecological coherence with regard to their salinity preferences. We found a tight coupling of microbial community structure and function, and demonstrated that salinity selects for organisms with phylogenetically-clustered salinity preferences. An influence of phylogeny was also detected within organisms that displayed no salinity preference, which suggests a genomic basis for this flexibility. Understanding how salinity changes will affect microbial communities and their associated functions is especially important given that climate change associated sea-level rise will expose many aquatic ecosystems to saltwater intrusion (Böning et al., 2008; Erwin, 2009; Nielsen and Brock, 2009; Helm et al., 2010).

### Response of Microbial Community Structure and Function to Altered Salinity

Our experimental manipulation of salinity led to a shift in the bacterial community, including a change in phylotype membership in the transplanted soils (**Figure 2A**). This is likely due, at least in part, to the colonization of organisms from the new host environment. Interestingly, few phylotypes disappeared from the transplanted soils relative to their origin controls, which could be interpreted as a widespread tolerance to the salinity change. However, a recent study by Logares et al. (2013) suggests bacteria are most often specialized with regard to their salinity habitat, making this possibility unlikely. A more probable explanation of our findings is that the change in salinity caused intolerant phylotypes to become dormant or die, and their residual DNA was detected in the *16S rRNA* gene library (Jones and Lennon, 2010; Morrissey et al., 2015).

When the relative abundance of phylotypes is considered along with these changes in community membership, it becomes clear that the origin of the soil was the dominant influence on microbial community composition (**Figure 2B**). These results are consistent with a scenario wherein organisms from the host environment have begun to colonize the transplanted soil (thus changing community membership) but have yet to establish large populations. This sort of gradual change in community composition is consistent with other salinity manipulations that have found saltwater to have little effect on community composition in the short term [∼1 month (Baldwin et al., 2006; Edmonds et al., 2009)] but significant impacts at longer time scales [months to years (Jackson and Vallaire, 2009; Berrier et al., 2012; Neubauer et al., 2012)]. A similar gradual change in community composition after transplantation into a new environment has been demonstrated in upland soil communities (Balser and Firestone, 2005). As with bacterial community composition, the dominant influence on microbial activity was the origin environment (**Table 1**). These results are consistent with past work showing that environmental history can have lasting effects on microbial community composition (Kulmatiski and Beard, 2011; Nelson et al., 2015) and function (Evans and Wallenstein, 2012; Peralta et al., 2013; Strickland et al., 2015). For instance, Nelson et al. (2015) found historical exposure to saltwater to be a greater driver of bacterial community composition than contemporary salinity. The strong relationship between bacterial community composition and activity rates in the present study suggests that the persistent effect of the origin environment on function could be mediated, at least in part, by microbial community composition [consistent with Reed and Martiny (2013)], which gradually shifts in response to changing environmental conditions. However, some functions, most notably CH_4_ production, responded to the change in salinity despite the relatively short incubation period. Reduced CH_4_ production following exposure to saltwater (**Table 1**) is consistent with other several other wetland studies (Weston et al., 2006; Chambers et al., 2011; Neubauer et al., 2013), and is likely due to sensitivity of the methanogen population to increased osmotic stress (Chambers et al., 2011) and/or competitive inhibition of methanogens by SRB (Poffenbarger et al., 2011). Our results support the latter hypothesis in that we saw a strong negative correlation between methanogen (*mcrA*) and SRB (*dsrA*) abundances across the treatments.

In addition to inhibiting methanogens, saltwater intrusion is expected to influence sulfate and iron-reducing bacteria. In this study, the abundance of δ-proteobacteria SRB was higher in the soils of saltwater origin (**Table 1**), as was the relative abundance of known SRB families (**Figure 2C**). This finding is consistent with past work that has found SRB to be abundant in saline marshes (Quillet et al., 2012; Angermeyer et al., 2015) and to co-vary with sulfate availability (Wilms et al., 2007), and provides support for the notion proposed by Oakley et al. (2010) that SRB vary in their salinity requirements. Taken together, our results support past research and suggest that SRB are likely to increase in abundance following saltwater intrusion with accompanying increases in sulfate reduction rates. These increased sulfate reduction rates may accelerate organic matter mineralization (Chambers et al., 2011; Weston et al., 2011, 2014; Neubauer, 2013), which could affect the ability of the wetland to sequester carbon and change the rate at which it emits greenhouse gasses (CO_2_).

Microbial iron reduction has been shown to be important in freshwater wetlands (Roden and Wetzel, 1996; Neubauer et al., 2005), though there have been very few studies that consider salinity effects (Neubauer and Craft, 2009; Schoepfer et al., 2014). Experimental increases in salinity have been shown to decrease iron reduction rates in wetland soils (Weston et al., 2006), perhaps due to abiotic interactions such as reactive iron removal due to pyrite formation (Berner, 1970), or possibly due to effects on iron reducing microorganisms. In our soils, the relative abundance of *Geobacter*, a putative FeRB, suggested intolerance of this taxa to saltwater (**Figure 2D**). This is consistent with pure culture studies that have found high salt concentrations to inhibit the growth of *Geobacter* species (Nevin et al., 2005; Sun et al., 2014). Further, *Geobacter* have been detected in a variety of freshwater environments (Caccavo et al., 1994; Coates et al., 1996; Nevin et al., 2005) and yet appear to be absent from the iron-reducing zone in salt marshes (Coleman et al., 1993) and marine sediments (Holmes et al., 2004). Interestingly, there are also reports of saltwater intrusion leading to increased iron reduction rates (Meiggs and Taillefert, 2011; Schoepfer et al., 2014), which are usually attributed to enhanced iron availability due to ionic effects like greater sediment deposition. Presumably these higher iron reduction rates involve FeRB taxa that are more tolerant of salinity stress, revealing another instance wherein a greater understanding of microbial community composition could enhance our ability to predict ecosystem function.

### Salinity Preferences and Phylogeny

Our results contribute to a growing body of evidence that despite gene loss, convergent evolution, and lateral gene transfer in bacteria (Doolittle, 1999; Snel et al., 2002; Boucher et al., 2003), phylogenetic groups can exhibit ecological coherence even at high levels of taxonomic organization (Philippot et al., 2010). To date, phylogeny has been associated with the habitats (Stegen et al., 2012), growth responses (Goldfarb et al., 2011; Placella et al., 2012), and ecological strategies (Evans and Wallenstein, 2014) of bacteria. Our work builds upon these previous studies by demonstrating that salinity preferences are strongly tied to phylogeny both across and near the tips of the phylogenetic tree (as evidenced by our SES-PD and NTI results respectively). Specifically, the NTI assesses whether traits are locally clustered within terminal clades of the phylogenetic tree (Webb, 2000), while the SES-PD test assesses clustering across the entire phylogenetic tree, including deep branches (Faith and Baker, 2006; Procheş et al., 2006). The highly significant and negative SES-PD indicates that salinity preference is clustered within bacterial phylogeny, and the significant NTI indicates this clustering is maintained to the terminal branches of the phylogenetic tree. Consequently, our results suggest that the seemingly ubiquitous co-variation of bacterial community composition with salinity could arise from ecological coherence of salinity preference within phylogenetic groups.

The mechanism of microbial evolutionary adaptation to varying salinity regimes is not yet clear. One obvious possibility is that the adaptations underlying salinity preference relate to osmotic stress. Though the ability of bacteria to osmoregulate appears to be ubiquitous (Neidhardt et al., 1990), taxa clearly vary in their physiological responses (Csonka, 1989; Romantsov et al., 2009). It also appears that salinity is linked to differences in key metabolic capabilities in bacteria, including large differences in the relative abundance of genes associated with respiration, glycolysis, biosynthesis of quinones, and osmolyte transport (Dupont et al., 2014). Dupont et al. (2014) argue that these striking differences in central metabolic processes suggest that adaptations to salinity regime emerged early during the divergence of phylogenetic groups in bacteria, a hypothesis consistent with our SES-PD results.

The taxonomic groups that exhibited a preference for fresh or saltwater in our soils were in accordance with patterns documented in other aquatic systems. For instance, we found β-proteobacteria to have a higher relative abundance in soil originating from the freshwater site (**Figure 1**) and a higher than expected preference for freshwater (Supplemental Figure S1). This is consistent with work by del Giorgio and Bouvier (2002) in an estuary and Herlemann et al. (2011) in the Baltic Sea, as both of these studies found the relative abundance of β-proteobacteria to decrease with increasing salinity. Also consistent with these studies, we found α- and γ-proteobacteria to have a higher than expected preference for saltwater. Taken together, these results support the findings of Lozupone and Knight (2007) in suggesting that salinity is a consistent driver of phylogenetic bacterial community structure across ecosystem types. The phylotypes that did not exhibit a strong salinity preference could be composed of ecologically differentiated subgroups that cannot be distinguished at 97% *16S rRNA* gene sequence identity (Jezbera et al., 2013). For instance, Jaspers and Overmann (2004) isolated 11 strains of *Brevundimonas alba* from a freshwater lake that had identical *16S rRNA* gene sequences but very little niche overlap. However, such rapid ecological diversification is inconsistent with the strong phylogenetic clustering we found in the “no salinity preference” group. A more parsimonious explanation is that these phylotypes are salinity generalists. Their survival under such discordant environmental conditions may reflect significant phenotypic plasticity (Snell-Rood et al., 2010), and the phylogenetic clustering we detected could indicate the genetic modules enabling this plasticity were conserved over evolutionary time.

Based on the phylogenetically-clustered salinity preferences documented here, we expect that saltwater intrusion will affect bacterial biodiversity by favoring organisms with a preference for saltwater over those with a preference for freshwater. For instance, saltwater intrusion may lead to a reduction in the abundance and diversity of β-proteobacteria and an increase in the abundance and diversity of α- and γ-proteobacteria. Such changes in biodiversity are likely to affect microbial activities such as EEA and soil respiration given the tight association between bacterial community composition and activity documented here and in other wetland studies (e.g., Peralta et al., 2010; Morrissey et al., 2014b).

This research provides evidence for ecological coherence with regard to the salinity preferences of bacterial phylogenetic groups, and suggests that global patterns in microbial biodiversity arise from divergent evolution deeply rooted in the phylogenetic tree. Most prior work has simply correlated changes in salinity with differences in community composition, and justified patterns based on contemporary physiological responses (e.g., a population’s tolerance of osmotic stress). Our study demonstrates that high levels of taxonomic organization can serve as ecologically meaningful units, and suggests that a more thorough consideration of an organism’s evolutionary history could help resolve several unanswered questions in microbial ecology. In particular, our work highlights the potential use of phylogenetic information for predicting the distribution of bacterial taxa along environmental gradients and for determining how environmental perturbations may shape community composition. As microbial ecologists gather this sort of information for an increasingly large number of environmental variables, we will develop a more robust understanding of microbial biogeography and become better able to predict bacterial community responses to global change.

## Author Contributions

EM and RF designed the research. EM performed the laboratory and data analyses. EM and RF wrote the paper.

## Conflict of Interest Statement

The authors declare that the research was conducted in the absence of any commercial or financial relationships that could be construed as a potential conflict of interest.

## References

[B1] Angermeyer A. Crosby S. C. Huber J. A. (2015). Decoupled distance-decay patterns between dsrA and 16S rRNA genes among salt marsh sulfate-reducing bacteria. *Environ. Microbiol.* 10.1111/1462-2920.12821 [Epub ahead of print]. 25727503

[B2] Baldwin D. S. Rees G. N. Mitchell A. M. Watson G. Williams J. (2006). The short-term effects of salinization on anaerobic nutrient cycling and microbial community structure in sediment from a freshwater wetland. *Wetlands* 26 455–464. 10.1672/0277-5212(2006)26[455:TSEOSO]2.0.CO;2

[B3] Balser T. C. Firestone M. K. (2005). Linking microbial community composition and soil processes in a California annual grassland and mixed-conifer forest. *Biogeochemistry* 73 395–415. 10.1007/s10533-004-0372-y

[B4] Bartram A. K. Lynch M. D. J. Stearns J. C. Moreno-Hagelsieb G. Neufeld J. D. (2011). Generation of multimillion-sequence 16S rRNA gene libraries from complex microbial communities by assembling paired-end Illumina reads. *Appl. Environ. Microbiol.* 77 3846–3852. 10.1128/AEM.02772-10 21460107 PMC3127616

[B5] Berner R. A. (1970). Sedimentary pyrite formation. *Am. J. Sci.* 268 1–23. 10.2475/ajs.268.1.1

[B6] Berrier D. J. Franklin R. B. Battistelli J. M. Neubauer S. C. (2012). “The effect of saltwater intrusion on microbial community structure and function in a tidal freshwater marsh,” in *Proceedings of the 9th INTECOL International Wetlands Conference*, Orlando, FL.

[B7] Bokulich N. A. Subramanian S. Faith J. J. Gevers D. Gordon J. I. Knight R. (2013). Quality-filtering vastly improves diversity estimates from Illumina amplicon sequencing. *Nat. Methods* 10 57–59. 10.1038/nmeth.2276 23202435 PMC3531572

[B8] Böning C. W. Dispert A. Visbeck M. Rintoul S. R. Schwarzkopf F. U. (2008). The response of the antarctic circumpolar current to recent climate change. *Nat. Geosci.* 1 864–869. 10.1038/ngeo362

[B9] Boucher Y. Douady C. J. Papke R. T. Walsh D. A. Boudreau M. E. R. Nesbø C. L. (2003). Lateral gene transfer and the origins of prokaryotic groups. *Annu. Rev. Genet.* 37 283–328. 10.1146/annurev.genet.37.050503.084247 14616063

[B10] Caccavo F. Lonergan D. J. Loveley D. R. Davis M. Stolz J. F. McInerney M. J. (1994). *Geobacter* sulfurreducens sp. nov., a hydrogen- and acetate-oxidizing dissimilatory metal-reducing microorganism. *Appl. Environ. Microbiol.* 60 3752–3759. 7527204 10.1128/aem.60.10.3752-3759.1994 PMC201883

[B11] Caporaso J. G. Kuczynski J. Stombaugh J. Bittinger K. Bushman F. D. Costello E. K. (2010). QIIME allows analysis of high-throughput community sequencing data. *Nat. Methods* 7 335–336. 10.1038/nmeth.f.303 20383131 PMC3156573

[B12] Cavender-Bares J. Kozak K. H. Fine P. V. A. Kembel S. W. (2009). The merging of community ecology and phylogenetic biology. *Ecol. Lett.* 12 693–715. 10.1111/j.1461-0248.2009.01314.x 19473217

[B13] Chambers L. G. Reddy K. R. Osborne T. Z. (2011). Short-term response of carbon cycling to salinity pulses in a freshwater wetland. *Soil Sci. Soc. Am. J.* 75 2000–2007. 10.2136/sssaj2011.0026

[B14] Coates J. D. Phillips E. J. P. Lonergan D. J. Jenter H. Lovely D. R. (1996). Isolation of *Geobacter* species from diverse sedimentary environments. *Appl. Environ. Microbiol.* 62 1531–1536. 8633852 10.1128/aem.62.5.1531-1536.1996 PMC167928

[B15] Coleman M. L. Hedrick D. B. Lovely D. R. White D. C. Pye K. (1993). Reduction of Fe(III) in sediments by sulphate-reducing bacteria. *Nature* 361 436–438.

[B16] Craft C. (2007). Freshwater input structures soil properties, vertical accretion, and nutrient accumulation of Georgia and U.S. *tidal marshes.* *Limnol. Oceanogr.* 52 1220–1230. 10.4319/lo.2007.52.3.1220

[B17] Csonka L. N. (1989). Physiological and genetic responses of bacteria to osmotic stress. *Microbiol. Rev.* 53 121–147. 2651863 10.1128/mr.53.1.121-147.1989 PMC372720

[B18] del Giorgio P. A. Bouvier T. C. (2002). Linking the physiologic and phylogenetic successions in free-living bacterial communities along an estuarine salinity gradient. *Limnol. Oceanogr.* 42 471–486. 10.4319/lo.2002.47.2.0471

[B19] Donoghue M. J. (2008). A phylogenetic perspective on the distribution of plant diversity. *Proc. Natl. Acad. Sci. U.S.A*. 105 11549–11555. 10.1073/pnas.0801962105 18695216 PMC2556411

[B20] Doolittle W. F. (1999). Phylogenetic classification and the universal tree. *Science* 284 2124–2128. 10.1126/science.284.5423.2124 10381871

[B21] Dupont C. L. Larsson J. Yooseph S. Ininbergs K. Goll J. Asplund-Samuelsson J. (2014). Functional tradeoffs underpin salinity-driven divergence in microbial community composition. *PLoS ONE* 9:e89549 10.1371/journal.pone.0089549 PMC3937345 24586863

[B22] Edgar R. C. (2010). Search and clustering orders of magnitude faster than BLAST. *Bioinformatics* 26 2460–2461. 10.1093/bioinformatics/btq461 20709691

[B23] Edmonds J. W. Weston N. B. Joye S. B. Mou X. Moran M. A. (2009). Microbial community response to seawater amendment in low-salinity tidal sediments. *Microb. Ecol.* 58 558–568. 10.1007/s00248-009-9556-2 19629578

[B24] Efron B. Tibshirani R. (1993). *An Introduction to the Bootstrap.* New York, NY: CRC Press LLC.

[B25] Erwin K. L. (2009). Wetlands and global climate change: the role of wetland restoration in a changing world. *Wetl. Ecol. Manag.* 17 71–84. 10.1007/s11273-008-9119-1

[B26] Ettema T. J. G. Andersson S. G. E. (2009). The alpha-proteobacteria: the Darwin finches of the bacterial world. *Biol. Lett.* 5 429–432. 10.1098/rsbl.2008.0793 19324639 PMC2679921

[B27] Evans S. E. Wallenstein M. D. (2012). Soil microbial community response to drying and rewetting stress: does historical precipitation regime matter? *Biogeochemistry* 109 101–116. 10.1007/s10533-011-9638-3

[B28] Evans S. E. Wallenstein M. D. (2014). Climate change alters ecological strategies of soil bacteria. *Ecol. Lett.* 17 155–164. 10.1111/ele.12206 24261594

[B29] Faith D. P. Baker A. M. (2006). Phylogenetic diversity (PD) and biodiversity conservation: some bioinformatics challenges. *Evol. Bioinform. Online* 2 121–128. 19455206 PMC2674678

[B30] Goldfarb K. C. Karaoz U. Hanson C. A. Santee C. A. Bradford M. A. Treseder K. K. (2011). Differential growth responses of soil bacterial taxa to carbon substrates of varying chemical recalcitrance. *Front. Microbiol.* 2:94 10.3389/fmicb.2011.00094 PMC3153052 21833332

[B31] Hammer Ø (2001). PAST: paleontological statistics software package for education and data analysis. *Palaeontol. Electronica* 4 1–9.

[B32] Helm K. P. Bindoff N. L. Church J. A. (2010). Changes in the global hydrological-cycle inferred from ocean salinity. *Geophys. Res. Lett.* 37:L18701 10.1029/2010GL044222

[B33] Herbert E. R. Boon P. Burgin A. J. Neubauer S. C. Franklin R. B. Ardón M. (2015). A global perspective on wetland salinization: ecological consequences of a growing threat to freshwater wetlands. *Ecosphere* (in press).

[B34] Herlemann D. P. R. Labrenz M. Jürgens K. Bertilsson S. Waniek J. J. Andersson A. F. (2011). Transitions in bacterial communities along the 2000 km salinity gradient of the Baltic Sea. *ISME. J.* 5 1571–1579. 10.1038/ismej.2011.41 21472016 PMC3176514

[B35] Holmes D. E. Nicoll J. S. Bond D. R. Lovely D. R. (2004). Potential role of a novel psychrotolerant member of the family Geobacteraceae, *Geopsychrobacter electrodiphilus* gen. nov., sp. nov., in electricity production by a marine sediment fuel cell. *Appl. Environ. Microbiol.* 70 6023–6030. 15466546 10.1128/AEM.70.10.6023-6030.2004 PMC522133

[B36] Hopfensperger K. N. Burgin A. J. Schoepfer V. A. Helton A. M. (2014). Impacts of saltwater incursion on plant communities, anaerobic microbial metabolism, and resulting relationships in a restored freshwater wetland. *Ecosystems* 17 792–807. 10.1007/s10021-014-9760-x

[B37] Jackson C. R. Vallaire S. C. (2009). Effects of salinity and nutrient enrichment on microbial assemblages in Louisiana wetland sediments. *Wetlands* 29 277–287. 10.1672/08-86.1

[B38] Jaspers E. Overmann J. (2004). Ecological significance of microdiversity: identical 16s rrna gene sequences can be found in bacteria with highly divergent genomes and ecophysiologies. *Appl. Environ. Microbiol.* 70 4831–4839. 15294821 10.1128/AEM.70.8.4831-4839.2004 PMC492463

[B39] Jezbera J. Jezberová J. Kasalický V. Šimek K. Hahn M. W. (2013). Patterns of *Limnohabitans* microdiversity across a large set of freshwater habitats as revealed by Reverse Line Blot Hybridization. *PLoS ONE* 2013 8:e58527 10.1371/journal.pone.0058527 PMC3595293 23554898

[B40] Jones S. E. Lennon J. T. (2010). Dormancy contributes to the maintenance of microbial diversity. *Proc. Natl. Acad. Sci. U.S.A.* 107 5881–5886. 10.1073/pnas.0912765107 20231463 PMC2851880

[B41] Kembel S. W. (2009). Disentangling niche and neutral influences on community assembly: assessing the performance of community phylogenetic structure tests. *Ecol. Lett.* 12 949–960. 10.1111/j.1461-0248.2009.01354.x 19702749

[B42] Kulmatiski A. Beard K. H. (2011). Long-term plant growth legacies overwhelm short-term plant growth effects on soil microbial community structure. *Soil Biol. Biochem.* 43 823–830. 10.1016/j.soilbio.2010.12.018

[B43] Langille M. G. Zaneveld J. Caporaso J. G. McDonald D. Knights D. Reyes J. A. (2013). Predictive functional profiling of microbial communities using 16S rRNA marker gene sequences. *Nat. Biotechnol.* 31 814–821. 10.1038/nbt.2676 23975157 PMC3819121

[B44] Logares R. Lindström E. S. Langenheder S. Logue J. B. Paterson H. Laybourn-Parry J. (2013). Biogeography of bacterial communities exposed to progressive long-term environmental change. *ISME. J.* 7 937–948. 10.1038/ismej.2012.168 23254515 PMC3635229

[B45] Lozupone C. Knight R. (2005). UniFrac: a new phylogenetic method for comparing microbial communities. *Appl. Environ. Microbiol.* 71 8228–8235. 10.1128/AEM.71.12.8228-8235.2005 16332807 PMC1317376

[B46] Lozupone C. A. Knight R. (2007). Global patterns in bacterial diversity. *Proc. Natl. Acad. Sci. U.S.A.* 104 11436–11440. 10.1073/pnas.0611525104 17592124 PMC2040916

[B47] Mahadevan R. Palsson B. Ø. Lovley D. R. (2011). In situ to in silico and back: elucidating the physiology and ecology of *Geobacter* spp. using genome-scale modelling. *Nat. Rev. Microbiol.* 9 39–50. 10.1038/nrmicro2456 21132020

[B48] McDonald D. Price M. N. Goodrich J. Nawrocki E. P. DeSantis T. Z. Probst A. (2012). An improved Greengenes taxonomy with explicit ranks for ecological and evolutionary analyses of bacteria and archaea. *ISME. J.* 6 610–618. 10.1038/ismej.2011.139 22134646 PMC3280142

[B49] Meiggs D. Taillefert M. (2011). The effect of riverine discharge on biogeochemical processes in estuarine sediments. *Limnol. Oceanogr.* 56 1797–1810. 10.4319/lo.2011.56.5.1797

[B50] Meyer F. Paarmann D. D’Souza M. Olson R. Glass E. M. Kubal M. (2008). The metagenomics RAST server–a public resource for the automatic phylogenetic and functional analysis of metagenomes. *BMC Bioinformatics* 9:386 10.1186/1471-2105-9-386 PMC2563014 18803844

[B51] Morrissey E. M. Berrier D. J. Neubauer S. C. Franklin R. B. (2014a). Using microbial communities and extracellular enzymes to link soil organic matter characteristics to greenhouse gas production in a tidal freshwater wetland. *Biogeochemistry* 117 473–490. 10.1007/s10533-013-9894-5

[B52] Morrissey E. M. Gillespie J. L. Morina J. C. Franklin R. B. (2014b). Salinity affects microbial activity and soil organic matter content in tidal wetlands. *Global Change Biol.* 20 1351–1362. 10.1111/gcb.12431 24307658

[B53] Morrissey E. M. McHugh T. A. Preteska L. Hayer M. Dijkstra P. Hungate B. A. (2015). Dynamics of extracellular DNA decomposition and bacterial community composition in soil. *Soil Biol. Biochem.* 86 42–49. 10.1016/j.soilbio.2015.03.020

[B54] Neidhardt F. C. Ingraham J. L. Schaechter M. (1990). *Physiology of the Bacterial Cell: A Molecular Approach.* Sunderland, MA: Sinauer Associates.

[B55] Nelson T. M. Streten C. Gibb K. S. Chariton A. A. (2015). Saltwater intrusion history shapes the response of bacterial communities upon rehydration. *Sci. Total Environ.* 502 143–148. 10.1016/j.scitotenv.2014.08.109 25247483

[B56] Neubauer S. C. (2013). Ecosystem responses of a tidal freshwater marsh experiencing saltwater intrusion and altered hydrology. *Estuaries Coasts* 36 491–507. 10.1007/s12237-011-9455-x

[B57] Neubauer S. C. Craft C. B. (2009). “Global change and tidal freshwater wetlands: scenarios and impacts,” in *Tidal Freshwater Wetlands*, eds Barendregt A. Whigham D. Baldwin A. (Weikersheim: Margraf Publishers), 253–265.

[B58] Neubauer S. C. Franklin R. B. Berrier D. J. (2013). Saltwater intrusion into tidal freshwater marshes alters the biogeochemical processing of organic carbon. *Biogeosciences* 10 8171–8183. 10.5194/bg-10-8171-2013

[B59] Neubauer S. C. Franklin R. B. Piehler M. F. (2012). “Saltwater intrusion into tidal freshwater marshes drives shifts at all levels of ecosystem organization,” in *Proceedings of the 9th INTECOL International Wetlands Conference*, Orlando, Florida.

[B60] Neubauer S. C. Givler K. Valentine S. K. Megonigal J. P. (2005). Seasonal patterns and plant-mediated controls of subsurface wetland biogeochemistry. *Ecology* 86 3334–3344. 10.1890/04-1951

[B61] Nevin K. P. Holmes D. E. Woodard T. L. Hinlein E. S. Ostendorf D. W. Lovley D. (2005). *Geobacter* bemidjiensis sp. nov. and *Geobacter psychrophilus* sp. nov., two novel Fe(III)-reducing subsurface isolates. *Int. J. Syst. Evol. Microbiol.* 55 1667–1674. 10.1099/ijs.0.63417-0 16014499

[B62] Nielsen D. L. Brock M. A. (2009). Modified water regime and salinity as a consequence of climate change: prospects for wetlands of Southern Australia. *Clim. Change* 95 523–533. 10.1007/s10584-009-9564-8

[B63] Oakley B. B. Carbonero F. van der Gast C. J. Hawkins R. J. Purdy K. J. (2010). Evolutionary divergence and biogeography of sympatric niche-differentiated bacterial populations. *ISME J.* 4 488–497. 10.1038/ismej.2009.146 20054357

[B64] Oksanen J. Blanchet F. G. Kindt R. Legendre P. Minchin P. R. O’Hara R. B. (2013). *Vegan: Community Ecology Package. R package Version 2.0-10.* Available at: http://CRAN.R-project.org/package=vegan

[B65] Paradis E. Claude J. Strimmer K. (2004). APE: analyses of phylogenetics and evolution in R language. *Bioinformatics* 20 289–290. 10.1093/bioinformatics/btg412 14734327

[B66] Peralta A. L. Ludmer S. Kent A. D. (2013). Hydrologic history influences microbial community composition and nitrogen cycling under experimental drying/wetting treatments. *Soil Biol. Biochem.* 66 29–37. 10.1016/j.soilbio.2013.06.019

[B67] Peralta A. L. Matthews J. W. Kent A. D. (2010). Microbial community structure and denitrification in a wetland mitigation bank. *Appl. Environ. Microbiol.* 76 4207–4215. 10.1128/AEM.02977-09 20453124 PMC2897436

[B68] Pereyra L. P. Hiibel S. R. Prieto Riquelme M. V. Reardon K. F. Pruden A. (2010). Detection and quantification of functional genes of cellulose-degrading, fermentative, and sulfate-reducing bacteria and methanogenic archaea. *Appl. Environ. Microbiol.* 76 2192–2202. 10.1128/AEM.01285-09 20139321 PMC2849247

[B69] Philippot L. Andersson S. G. Battin T. J. Prosser J. I. Schimel J. P. Whitman W. B. (2010). The ecological coherence of high bacterial taxonomic ranks. *Nat. Rev. Microbiol.* 8 523–529. 10.1038/nrmicro2367 20531276

[B70] Placella S. A. Brodie E. L. Firestone M. K. (2012). Rainfall-induced carbon dioxide pulses result from sequential resuscitation of phylogenetically clustered microbial groups. *Proc. Natl. Acad. Sci. U.S.A.* 109 10931–10936. 10.1073/pnas.1204306109 22715291 PMC3390866

[B71] Procheş S. Wilson J. R. Cowling R. M. (2006). How much evolutionary history in a 10 × 10 m plot? *Proc. R. Soc. B-Biol. Sci.* 273 1143–1148. 10.1098/rspb.2005.3427 PMC1560258 16600893

[B72] Poffenbarger H. J. Needelman B. A. Megonigal J. P. (2011). Salinity influence on methane emissions from tidal marshes. *Wetlands* 31 831–842. 10.1007/s13157-011-0197-0

[B73] Quillet L. Ludovic B. Popova M. Paissé S. Deloffre J. Ouddane B. (2012). Abundance, diversity and activity of sulfate-reducing prokaryotes in heavy metal-contaminated sediment from a salt marsh in the Medway Estuary (UK). *Mar. Biotechnol.* 14 363–381. 10.1007/s10126-011-9420-5 22124626

[B74] Reed H. E. Martiny J. B. H. (2013). Microbial composition affects the functioning of estuarine sediments. *ISME J.* 7 868–879. 10.1038/ismej.2012.154 23235294 PMC3603390

[B75] Roache M. C. Bailey P. C. Boon P. I. (2006). Effects of salinity on the decay of the freshwater macrophyte. *Triglochin* procerum. *Aquat. Bot.* 84 45–52. 10.1016/j.aquabot.2005.07.014

[B76] Roden E. E. Wetzel R. G. (1996). Organic carbon oxidation and suppression of methane production by microbial Fe(III) oxide reduction in vegetated and unvegetated freshwater wetland sediments. *Limnol. Oceanogr.* 41 1733–1748. 10.4319/lo.1996.41.8.1733

[B77] Romantsov T. Guan Z. Wood J. M. (2009). Cardiolipin and the osmotic stress responses of bacteria. *Biochim. Biophys. Acta* 1788 2092–2100. 10.1016/j.bbamem.2009.06.010 19539601 PMC3622477

[B78] Sall J. Creighton L. Lehman A. (2005). *JMP Start Statistics: A Guide to Statistics and Data Analysis Using JMP and JMP in Software.* Cary, NC: SAS Institute Inc.

[B79] Schoepfer V. Bernhardt E. S. Burgin A. J. (2014). Iron clad wetlands: soil iron-sulfur buffering determines coastal wetland response to salt water incursion. *JGR Biogeosci.* 119 2209–2219.

[B80] Silvertown J. Dodd M. Gowing D. Lawson C. McConway K. (2006). Phylogeny and the hierarchical organization of plant diversity. *Ecology* 87 S39–S49. 10.1890/0012-9658(2006)87[39:PATHOO]2.0.CO;2 16922301

[B81] Sinsabaugh R. L. Saiya-Cork K. Long T. Osgood M. P. Neher D. A. Zak D. R. (2003). Soil microbial activity in a Liquidambar plantation unresponsive to CO_2_-driven increases in primary production. *Appl. Soil. Ecol.* 24 263–271. 10.1016/S0929-1393(03)00002-7

[B82] Snel B. Bork P. Huynen M. A. (2002). Genomes in flux: the evolution of archaeal and proteobacterial gene content. *Genome Res.* 12 17–25. 10.1101/gr.176501 11779827

[B83] Snell-Rood E. C. Van Dyken J. D. Cruickshank T. Wade M. J. Moczek A. P. (2010). Toward a population genetic framework of developmental evolution: the costs, limits, and consequences of phenotypic plasticity. *Bioessays* 32 71–81. 10.1002/bies.200900132 20020499 PMC3151734

[B84] Stegen J. C. Lin X. Konopka A. E. Fredrickson J. K. (2012). Stochastic and deterministic assembly processes in subsurface microbial communities. *ISME J.* 6 1653–1664. 10.1038/ismej.2012.22 22456445 PMC3498916

[B85] Strickland M. S. Keiser A. Bradford M. A. (2015). Climate history shapes contemporary leaf litter decomposition. *Biogeochemistry* 122 165–174. 10.1007/s10533-014-0065-0

[B86] Sun D. Call D. Wang A. Cheng S. Logan B. E. (2014). *Geobacter* sp. *SD*-1 with enhanced electrochemical activity in high-salt concentration solutions. *Envrion. Microbiol. Rep.* 6 723–729. 10.1111/1758-2229.12193 25756125

[B87] Vasi F. Travisano M. Lenski R. E. (1994). Long-term experimental evolution in *Escherichia coli*. II. Changes in life-history traits during adaptation to a seasonal environment. *Am. Nat.* 144 432–456.

[B88] Webb C. O. (2000). Exploring the phylogenetic structure of ecological communities: an example for rain forest trees. *Am. Nat.* 156 145–155. 10.1086/303378 10856198

[B89] Weiss R. (1974). Carbon dioxide in water and seawater: the solubility of a non-ideal gas. *Mar. Chem.* 2 203–215. 10.1016/0304-4203(74)90015-2

[B90] Weston N. B. Dixon R. E. Joye S. B. (2006). Ramifications of increased salinity in tidal freshwater sediments: geochemistry and microbial pathways of organic matter mineralization. *J. Geophys. Res.* 111 G01009.

[B91] Weston N. B. Neubauer S. C. Velinsky D. J. Vile M. A. (2014). Net ecosystem carbon exchange and the greenhouse gas balance of tidal marshes along an estuarine salinity gradient. *Biogeochemistry* 120 163–189. 10.1007/s10533-014-9989-7

[B92] Weston N. B. Vile M. A. Neubauer S. C. Velinsky D. J. (2011). Accelerated microbial organic matter mineralization following salt-water intrusion into tidal freshwater marsh soils. *Biogeochemistry* 102 135–151. 10.1007/s10533-010-9427-4

[B93] Wilkinson L. (2012). Exact and approximate area-proportional circular Venn and Euler diagrams. *IEEE Trans. Vis. Comput. Graph.* 18 321–331. 10.1109/TVCG.2011.56 21383412

[B94] Wilms R. Sass H. Köpke B. Cypionka H. Engelen B. (2007). Methane and sulfate profiles within the subsurface of a tidal flat are reflected by the distribution of sulfate-reducing bacteria and methanogenic archaea. *FEMS Microbiol. Ecol.* 59 611–621. 10.1111/j.1574-6941.2006.00225.x 17059478

[B95] Zhou J. He Q. Hemme C. L. Mukhopadhyay A. Hillesland K. Zhou A. (2011). How sulphate-reducing microorganisms cope with stress: lessons from systems biology. *Nat. Rev. Microbiol.* 9 452–466. 10.1038/nrmicro2575 21572460

