# Supplementary material for: Evolutionary history influences the salinity preference of bacterial taxa in wetland soils
Source: Front Microbiol. 2015 Oct 2;6:1013. doi: 10.3389/fmicb.2015.01013 (PMC4591843; doi:10.3389/fmicb.2015.01013)
Supplement: Supplementary file 1 [file Image_1.PDF]

Supplementary Material

Evolutionary history influences the salinity preference of bacterial taxa in wetland soils

Ember M. Morrissey[1,2] and Rima B. Franklin[1*]

[1]Laboratory of Microbial Ecology, Department of Biology, Virginia Commonwealth University, Richmond, VA, USA
[2]Center for Ecosystem Science and Society, Northern Arizona University, Flagstaff, AZ, USA

*** Correspondence:** Rima B. Franklin, Laboratory of Microbial Ecology, Department of Biology, Virginia Commonwealth University, 1000 West Cary Street, Richmond, VA, 23284, USA
rbfranklin@vcu.edu

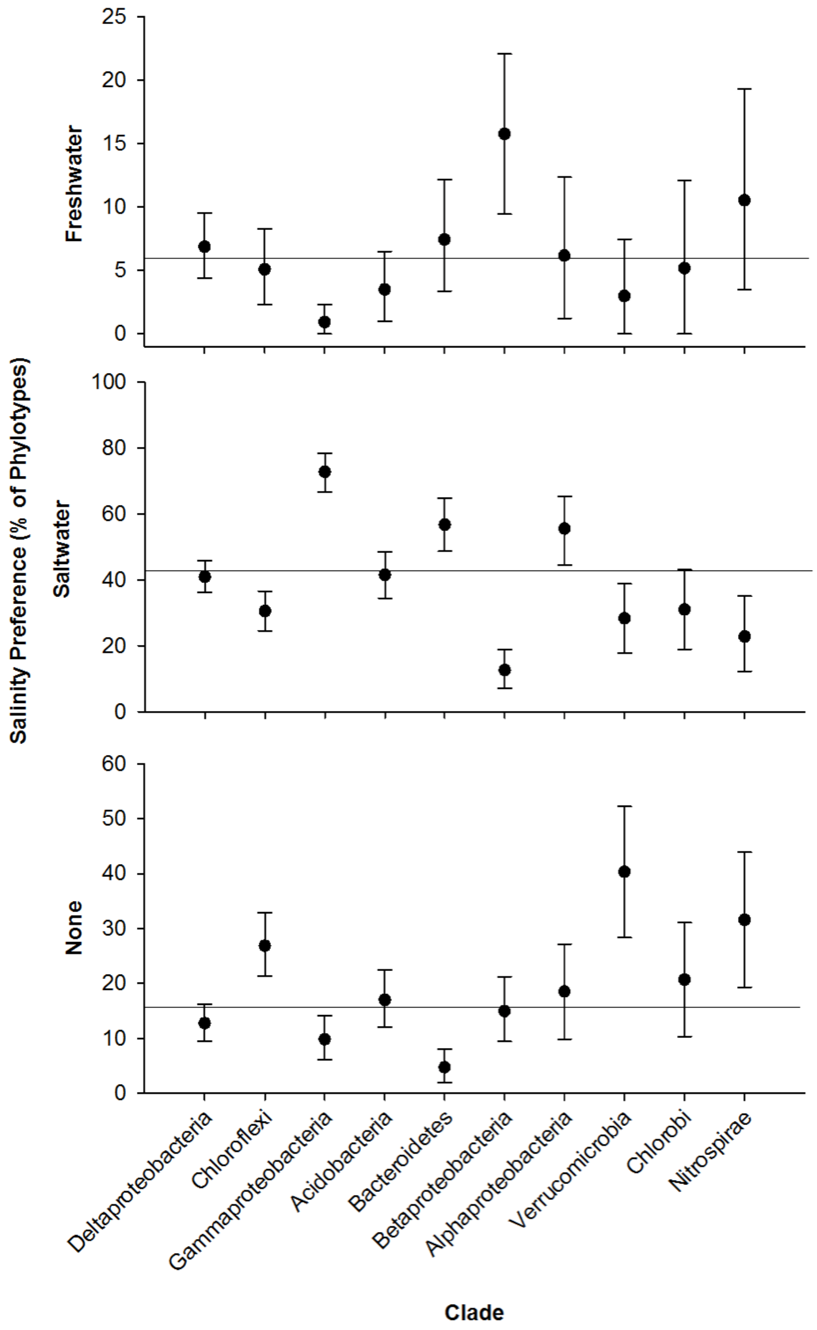

Supplemental Figure 1. Proportion of phylotypes within each salinity preference across the dominant phylogenetic clades. Data are means ± 95% confidence intervals produced from bootstrapping. Each line represent the expected random value (% of total phylotypes in each preference category), a deviation from random indicates a tendency for or against the preference.
